# Selection of a breast cancer subpopulation-specific antibody using phage display on tissue sections

**DOI:** 10.1007/s12026-015-8657-x

**Published:** 2015-05-12

**Authors:** Simon Asbjørn Larsen, Theresa Meldgaard, Agla J. Fridriksdottir, Simon Lykkemark, Pi Camilla Poulsen, Laura F. Overgaard, Helene Bundgaard Petersen, Ole William Petersen, Peter Kristensen

**Affiliations:** Department of Molecular Biology and Genetics, Aarhus University, Aarhus, Denmark; Department of Engineering, Aarhus University, Aarhus, Denmark; Department of Cellular and Molecular Medicine, Centre for Biological Disease Analysis and Danish Stem Cell Centre, University of Copenhagen, Copenhagen, Denmark; Department of Clinical Medicine, Aarhus University, Aarhus, Denmark; Sino-Danish Centre for Education and Research (SDC), Aarhus, Denmark

**Keywords:** Phage display, Biomarkers, Breast cancer, Tissue, CD271^+^, Shadow stick

## Abstract

Breast cancer tumors are composed of heterogeneous cell populations. These populations display a high variance in morphology, growth and metastatic propensity. They respond differently to therapeutic interventions, and some may be more prone to cause recurrence. Studying individual subpopulations of breast cancer may provide crucial knowledge for the development of individualized therapy. However, this process is challenged by the availability of biomarkers able to identify subpopulations specifically. Here, we demonstrate an approach for phage display selection of recombinant antibody fragments on cryostat sections of human breast cancer tissue. This method allows for selection of recombinant antibodies binding to antigens specifically expressed in a small part of the tissue section. In this case, a CD271^+^ subpopulation of breast cancer cells was targeted, and these may be potential breast cancer stem cells. We isolated an antibody fragment LH 7, which in immunohistochemistry experiments demonstrates specific binding to breast cancer subpopulations. The selection of antibody fragments directly on small defined areas within a larger section of malignant tissue is a novel approach by which it is possible to better target cellular heterogeneity in proteomic studies. The identification of novel biomarkers is relevant for our understanding and intervention in human diseases. The selection of the breast cancer-specific antibody fragment LH 7 may reveal novel subpopulation-specific biomarkers, which has the potential to provide new insight and treatment strategies for breast cancer.

**Electronic supplementary material:**

The online version of this article (doi:10.1007/s12026-015-8657-x) contains supplementary material, which is available to authorized users.

## Introduction

Breast cancer is characterized as being a very heterogeneous disease [1]. Effective detection, diagnosis and treatment are challenged by this heterogeneity. The heterogeneity in breast cancer greatly affects morphology, growth rate, metastatic propensity, therapeutic resistance and recurrence [2]. Breast cancer heterogeneity exists both between tumors as different subtypes (inter-tumor heterogeneity) and within a given tumor (intra-tumor heterogeneity). Multiple molecular subtypes among patients have been identified, each differing with respect to prevalence, prognosis and approach of treatment. The five most commonly used classifications to account for the inter-tumor heterogeneity include the luminal A, luminal B, basal-like, HER 2 and normal-like subtypes [3–6]. These subtypes mainly differ in the expression of the estrogen, progesterone, ErbB2 receptors and certain cytokeratins. Breast cancer is also characterized by intra-tumor heterogeneity. Therefore, multiple biopsies taken from the same tumor may reveal profound genomic variations, which indicate the presence or absence of different subpopulations within the tumor [7]. Two proposed models have hypothesized the cause for intra-tumor heterogeneity. According to the theory of clonal evolution, the genetic variations in the cells of a primary tumor are under selective pressure to adapt to their particular microenvironment [8]. These cells accumulate genetic and epigenetic alterations over time, and the selection pressure drives the evolution. The most adaptive cells become responsible for tumor progression and may form increasingly malignant, metastatic or drug-resistant tumors. This concept is supported by studies showing that metastatic potential coincides with genetic instability [9]. Another hypothesis involves cancer stem cells (CSC), which were initially identified in acute myeloid leukemia, but have since been reported in a wide variety of cancers [10]. This particular subpopulation may initiate tumors but also possess the ability to self-renew by either symmetric or asymmetric cell division, and they are capable of forming differentiation hierarchies within the tumor [11]. This differentiation generates heterogeneous cell lineages which constitute the bulk of the tumor and play different roles in metastasis, tumor recurrence and drug resistance. Today, it is becoming increasingly accepted that these two models are not mutually exclusive. Studies on leukemia stem cells have also shown that these particular CSCs may undergo clonal evolution themselves [12]. Hence, different breast cancer subpopulations occurring within the same tumor may be the result of both clonal evolution and CSCs [13]. Another consideration is that intra-tumor heterogeneity is not only shaped by intrinsic factors, but also by the complex network of cellular interactions with the microenvironment in which the tumor resides. Surrounding stroma cells, extracellular matrix, paracrine factors or local conditions such as hypoxia have all been shown to influence tumor progression [14–17]. The microenvironment may also influence therapeutic response [18]. Thus, the unique characteristics of an individual tumor derive from a combination of both intra-tumor heterogeneity and tumor microenvironment.

Here, we utilize phage display technology on cryostat breast cancer tissue to generate recombinant antibody fragments that specifically recognize subpopulations. We apply a method, which allows selections to be targeted at small subpopulations of primary breast cancer cells in tissue [19]. The use of cryostat tissue as biological material allows inclusion of the tumor microenvironment in the selections, which may provide antibody fragments of high clinical relevance. Selection was performed against CD271^+^ breast cancer cells directly on the tissue. These cells not only have the ability to initiate tumors, but are also capable of forming a differentiation hierarchy [20].

In this study, we demonstrate the selection of the antibody fragment LH 7, which bind an antigen expressed by certain breast cancer subpopulations. The discovery of LH 7 may allow further characterization of breast cancer subpopulations and bring new insights to the breast cancer field.

## Materials and methods

### Tissue sections

Cryostat sections (6–8 µm) from snap-frozen biopsies of breast cancer patients and healthy donors were prepared as described [21]. The use of human material has been reviewed by the Regional Scientific Ethical Committees (Region Hovedstaden) and approved with reference to H-2-2011-052 and H-2-2010-051. Tissue sections used for selections were fixed for 10 min in 3.7 % formaldehyde (Sigma-Aldrich), washed in PBS and incubated twice for 7 min in 0.01 % Triton X-100 (Sigma-Aldrich). Nuclei were counterstained with hematoxylin (Sigma-Aldrich). Selections were performed on basal-like breast cancer tissue from patient 757 [22] with the marker profile: (ER/PR^-/-^, cytokeratin (CK)17^+^, CK5^+^, low ErbB2, MM^+^ and CD271^+^). Tissue sections used for immunohistochemistry (IHC) were fixed in ice-cold methanol (Sigma-Aldrich) at −20 °C for 5 min. Tissue sections from four breast cancer patients were used, two basal-like breast cancers: P757 and P918 (ER^−^, CK17^+^, CK5^+^, MM^−^ and CD271^+^) and two luminal breast cancers: P761 (ER/PR^+/+^, CK17^−^, CK5^+^, low ErbB2, MM^+^ and CD271^+^) and P686 (ER/PR^+/+^, CK17^−^, CK5^−^, low ErbB2, MM^+^ and CD271^+^).

### Target area identification by immunoperoxidase staining with anti-CD271

Briefly, multiple sections were cut. The middle section was methanol-fixed and used for immunoperoxidase, while the other sections were formalin-fixed as described. The tissue from the middle section was encircled with a PAP pen liquid blocker and blocked for 5 min in Ultra V Block (TA-060-UB, Thermo scientific). The tissue was incubated for 1 h with 50 µL mouse anti-p75 NGF receptor antibody (anti-CD271) [ME20.4] 1:50 (abcam, #ab8877), washed three times with PBS (Ca^2+^ and Mg^2+^ free) and then incubated with 50 µL Ultravision ONE HRP Polymer (Thermo Scientific) for 30 min, washed three times with PBS and finally incubated with 1 mg/mL 3′,3′-diaminobenzidine tetrahydrochloride (DakoCytomation) in PBS with freshly added 1 µL/mL of 30 % H_2_O_2_ (Sigma-Aldrich) for 10 min. The tissue slide was washed with PBS and distilled water before the nuclei were counterstained with hematoxylin (Sigma-Aldrich). CD271^+^ cancer cells were found within a cancer nest in the methanol-fixed section. Corresponding areas were identified on the formalin sections and used for shadow stick selection.

### Shadow stick

The shadow sticks were fabricated from pulled injection microcapillaries (Tritech research, USA). The metal disks were made by compressing sinter metal powder, kindly provided by Dansk Sintermetal (Denmark). The flat pieces of powdered metal were placed on a microscope slide and attached to the tip of a pulled glass capillary by drawing up a small volume of epoxy glue into the capillary. Subsequently, the glue was dispensed on top of a piece of metal with a desired size. This procedure was done with the capillary attached to the micromanipulator, to ensure the disk being glued to the stick in the correct angle. This allows positioning of the disk on top of the target area using micromanipulation equipment from Narishige (Model MM-188, Nikon, Tokyo, Japan). A shadow stick with a diameter of approximately 150 µm was used in this study.

### Shadow stick selection of antibody fragments using phage display

The breast cancer tissue sections from P757 were formalin-fixed and blocked 1 h in 4 % Marvel dried skimmed milk powder (MPBS). The tissue slide was incubated with the phage library in a slide container containing 20 mL 2 % MPBS overnight with gentle agitation. The single-domain library “predator” was used which has diversity of 6.2 × 10^7^ different antibody fragments and a titer of 10^13^ pfu/mL with a display level estimated to be 6.4 % [23]. Fifty microliters phage stock was used per tissue section and incubated overnight. The slide was washed 10 min in PBS and two times 10 min in PBS with 10 % glycerol (PBSG) with gentle agitation. The slide was dried except from the target area, which was kept moist with approximately 10 µL PBSG. Using brightfield microscopy, the shadow stick was positioned above the target area. The slide was exposed to UV-C light (254 nm) for 5 min using a UV-C source (model UVSL-14P from UVP, Upland, CA, USA) positioned on a stand approximately 4 cm above the slide. Phage particles bound to the target area was eluted with 15 µL trypsin (1 mg/mL) for 15 min. Trypsin was aspirated and transferred to a tube before the area was washed 15 times with 50 µL PBSG, which was transferred to the eluate as well. For trypsin inactivation, 50 µL fetal bovine serum was added to the eluate before storage at −20 °C.

### Cell cultures

Myoepithelial cells were isolated from trypsinized organoids derived from normal breast tissue as previously described [24]. The trypsinized cells were incubated with fluorescent monoclonal anti-NGFR (neurotrophin receptor, p75)/CD271-APC (ME20.4, 1:50, Cedarlane Laboratories) for 45 min at 4 °C and then washed 2x in HEPES buffer supplemented with 0.5 % BSA (bovine fraction V; Sigma-Aldrich) and 2 mM EDTA (Merck) and finally incubated with 1 μg/mL propidium iodide (Life Technologies) to separate live from dead cells before analysis and sorting, using a FACSAria I flow cytometer (BD Biosciences). Sorted cells were set up in culture on collagen-coated flasks with BBMYAB medium. Establishment and culture of the CD271^+^ subclone from the BT474 cancer cell line were performed as described [20].

### Phage ELISA for screening and titration assay

Phage infection of *E*. *coli* and the production of phage antibodies were performed as described [19]. Initial screening of potential interesting phage antibodies was performed on CD271^+^ cancer cells. Titration assay was performed simultaneously on both CD271^+^ cancer cells and normal CD271^+^ myoepithelial cells, which enabled the possibility to reject the phage antibodies binding to common antigens. The assays were performed as described [19]. As a positive control for the phage ELISA procedure, the phage antibody 52 was used [25]. As a negative control, a phage antibody specific against fetal epsilon-hemoglobin was included [26]. For titration assay, phage antibodies of interest were produced in 50 mL TG-1 cultures and tested along with the above-mentioned controls in series of five fourfold serial dilutions, ranging from 10^11^ phages/well to 3.9 × 10^8^ as described [19]. Phage particles were quantified by measuring absorbance at 269 nm and 320 nm [23].

### Expression and purification of soluble antibody fragments

To express the individual clones as soluble antibody fragments, they were sub-cloned from the predator phagemid into a modified pET22b vector including c-Myc- and His-tag using *NcoI* and *NotI* restriction enzymes (Thermo Scientific) and T4 DNA ligase (Fermentas) before transformation into *BL21 Gold* (Agilent Technologies). Expression was initiated with a 4 mL overnight culture in TB medium containing ampicillin (100 µg/mL) and glucose (4 % w/v). The cultures were diluted 1:100 in 250 mL cultures and grown until OD_600_ of 0.6–0.8 and then spun for 10 min at 4 °C and 4000 rpm. The pellet was re-suspended into TB medium containing ampicillin (100 µg/mL) and IPTG (100 µg/mL) for induction and grown 16–18 h at 30 °C and 200 rpm. The cultures were spun for 1 h at 5000 g at 4 °C, and the antibody fragments in the supernatant was precipitated with 30 % w/v ammonium sulfate by incubation on a roller table at 4 °C overnight. The flasks were spun for 30 min at 5000 g at 4 °C and the pellet re-suspended in 40 mL TBS (pH 8) with approximately 400 U DNase I (Roche) including 5 mM Mg^+^. The solution was sterile-filtered with 0.20 µm filters (GF prefilters) and purified on HiTrap Protein A HP columns (GE Healthcare). The fractions containing the antibody fragments were determined by SDS-PAGE, pooled into a 3.5 kDa MW dialysis tube (Spectrum Laboratories) and dialyzed in 3 L TBS pH 7.5 at 4 °C overnight with gentle agitation. The dialyzed protein was transferred to 3 kDa MW VivaSpin columns (GE Healthcare) and spun down to a concentration of about 1 mg/mL measured on a NanoDrop spectrophotometer (Thermo Fisher Scientific). Purity was verified by SDS-PAGE and Western blotting against c-Myc.

### Immunohistochemical staining with soluble domain antibody fragments

The tissue sections were prepared as earlier described. The tissue was encircled with a PAP pen and blocked for 1 h with Ultra V Block (TA-060-UB, Thermo scientific). Approximately 25 µg antibody fragments were dissolved in Ultra V Block, 10 % goat serum and 1:100 anti-CK19 to a total volume of 100 µL and added to the encircled area. Incubation was performed for 3 h in humid chambers. The liquid was removed by aspiration, and the slide was washed four times 1 min in PBS. The slide was incubated for 30 min in the dark with mouse Cy3-conjugated anti-c-Myc antibody [9E10] 1:250 (Sigma-Aldrich), Alexa Fluor 488-conjugated goat anti-mouse IgG2a 1:500 (Invitrogen), DAPI 1:1000 (Invitrogen) and 10 % goat serum dissolved in Ultra V Block to 100 µL. Alternatively, the antibody fragments above were replaced with an anti-Ki67 antibody (Abcam) in a dilution of 1:100, and as secondary antibody, a goat anti-rabbit antibody coupled to Alexa 546 (Life technologies) was added in dilution of 1:500. The slide was washed three times 1 min in PBS and mounted with Fluoromount mounting media (Sigma-Aldrich) and cover glass.

## Results

### Outline

**Fig. 1:**
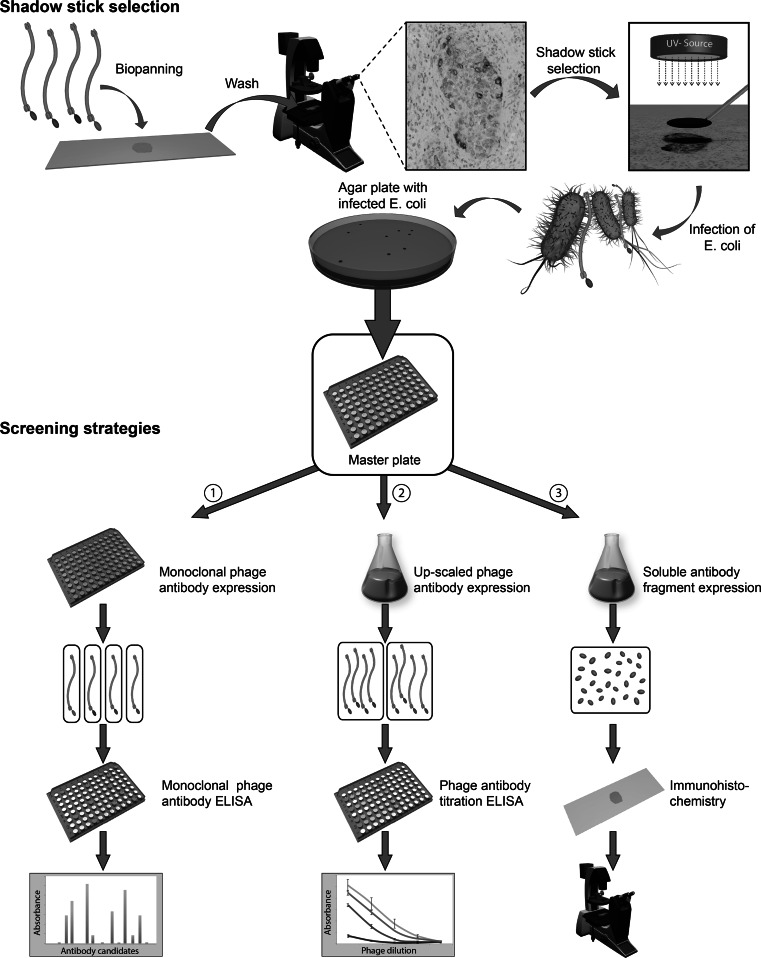
Illustration of the antibody fragment selection procedure and screening strategies. The target area for selection was chosen to be the middle part of a particular cancer nest due to clustered CD271^+^ staining present here. The entire tissue on a consecutive formalin-fixed section was then incubated with a phage library. The target area was relocated, and a minute disk (shadow stick) was positioned precisely above the target cells of interest. The target area was kept moist at all times. The shadow stick shielded the phage antibodies binding to the cells of interest from UV-C irradiation. The phages were eluted, but only those protected by the shadow stick can replicate in bacteria and provide ampicillin resistance. Each colony represented an antibody fragment, which required screening for their specificity. They were picked and grown in separate wells of a master plate. (1) In the initial screening, all colonies were grown in microtiter plates and monoclonal phage antibodies were produced. The phage antibodies were tested by phage ELISA on CD271^+^ cancer cells. (2) All phage antibodies binding with higher affinities than the negative control in the initial screening were produced monoclonally in 50 mL cultures. These were tested in different concentrations by a phage ELISA titration assay, which was performed simultaneously on CD271^+^ cancer cells and CD271^+^ myoepithelial cells. This provided comparative results of each phage antibody. (3) Soluble antibody fragments were expressed and purified and examined by IHC experiments on four different breast cancer biopsies to validate their specificity

The shadow stick selection procedure on cryostat tissue sections is based on our previous published work [19]. An outline of the selection and screening approach applied in this study can be observed in Fig. 1. The selections were performed with a novel single-domain phage antibody library termed “predator” [23].

### Identifying the target area

Selections were performed on multiple sections from a single biopsy (P757) which had small well-defined tumor cell nests and rare CD271^+^ staining throughout the tissue. The target area was chosen to be a high-density cluster of CD271^+^ cells within a single cancer nest (Fig. 1). The shadow stick shielded roughly 75–100 cells. Structural changes to the presentation of antigens might occur in the multiple steps of the CD271 immunostaining. Therefore, the selection was performed on separate, but consecutive non-stained tissue sections in order to preserve the antigens to the best ability. The consecutive slides were formalin-fixed and used fresh, and the target area was kept moist under all steps of the selection procedure. The exact corresponding target area was easily identified on the neighboring slides by the unique morphology patterns of the tumor cell nests.

### Selections and screening

Thirteen selections were performed on cryostat sections from the breast cancer patient 757 using the shadow stick on the target area as described; 315 clones were initially screened by phage ELISA on CD271 expressing cancer cells with phage antibodies produced in 96-well format. The phage antibody “epsilon” is specific against epsilon-hemoglobin almost exclusively expressed by fetal erythroblasts [26]. This phage antibody was included as negative control in the initial ELISA screening. Its absorbance value was chosen as a cutoff value to determine which of the 315 clones should be further analyzed. The initial ELISA screen serves the sole purpose of prioritizing clones for further analysis. The screening yielded 35 phage antibodies with higher absorbance compared with the negative control epsilon. The selection outputs of the predator library were generally of good quality, and sequencing of the individual genes encoding these 35 antibodies of interest revealed no stop codons, truncations, frameshift mutations or other abnormalities as expected. The 35 phage antibodies were produced in 50 mL cultures and tested by titration phage ELISA in series of five fourfold serial dilutions, ranging from 10^11^ phages/well to 3.9 × 10^8^ phages/well. This was performed simultaneously on both CD271^+^ cancer cells and CD271^+^ myoepithelial cells. This allows for comparison between the individual phage antibodies and the two cell lines. The phage antibodies binding equally well or better to myoepithelial cells were considered as common epitope binders and not tested further. Eleven out of the 35 tested phage antibodies were prioritized for further validation as they bound better to the CD271^+^ cancer cells compared to the CD271^+^ myoepithelial cells. Soluble antibody fragments were expressed and purified before validation by IHC.

### Test of antibody specificity by immunohistochemistry

**Fig. 2:**
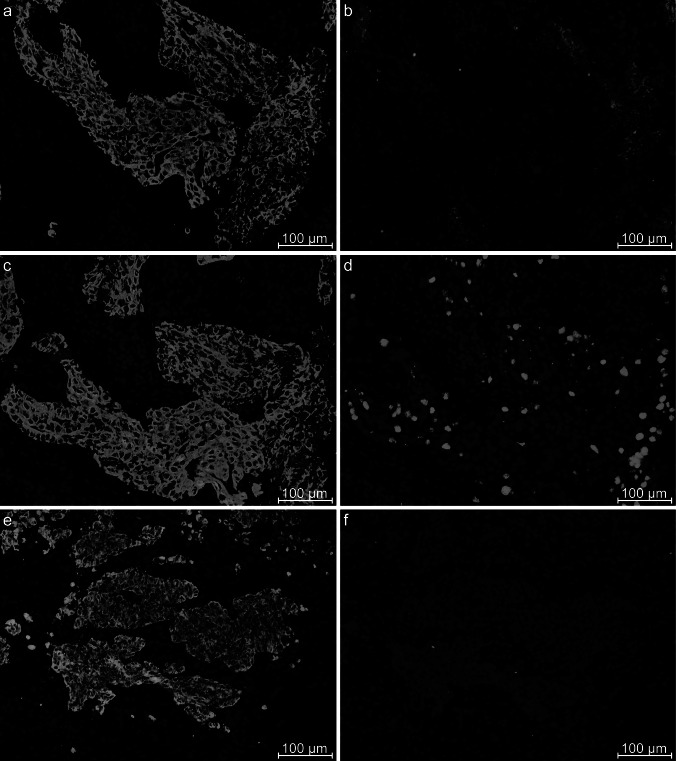
Immunohistochemistry on three separate sections of cryostat tissue from a basal-like breast cancer of patient 757, the same biopsy as selected upon. All pictures are merged with DAPI staining. The pictures **a**, **c** and **e** show staining against CK19, which indicate the presence of cancer cells. Picture **b** shows staining with the antibody fragment LH 7 which consistently only binds to a subset of cancer cells. The presence of cancer cells is confirmed by the staining with the proliferation marker ki67 within these areas as observed in picture **d**. Pictures **e** and **f** are from a different area than picture **a**–**d**, but it is the same biopsy. Picture **f** shows staining with the mouse Cy3-conjugated anti-c-Myc antibody used for detection of the antibody fragments. This shows that the observed staining is not caused by upregulated c-Myc expression in the cancer cells or unspecific binding by this secondary antibody

**Fig. 3:**
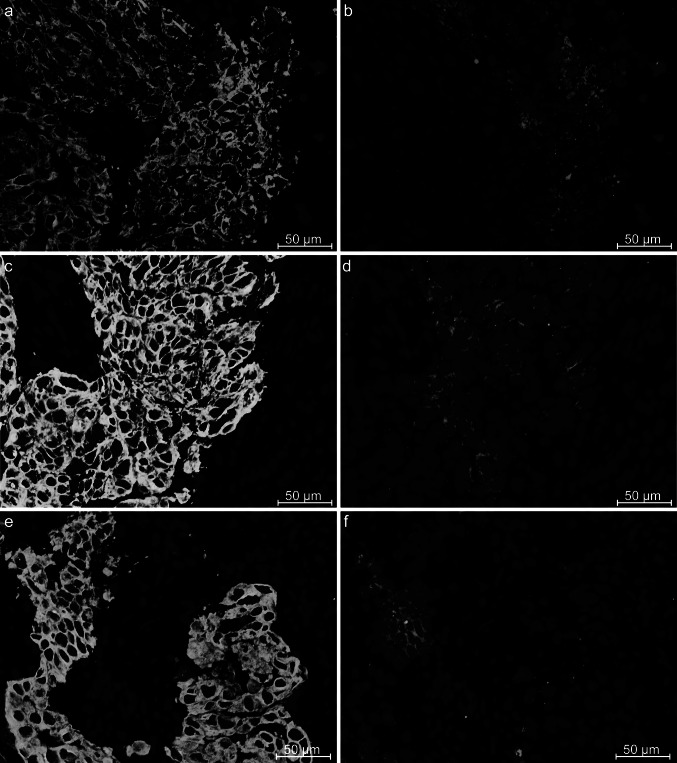
Immunohistochemistry showing three different areas on the same section of a cryostat tissue from a basal-like breast cancer of patient 757. All pictures are merged with DAPI staining. The pictures **a**, **c** and **e** show staining against CK19. Picture **b** shows staining with the antibody fragment LH 7 and is the exact same area as presented in Fig. 2b, but at higher magnification. Pictures **d** and **f** are LH 7 staining from other areas. Although all the pictures are from the same biopsy, there are slight variations in the staining pattern. Picture **f** shows more condensed or grouped staining compared with the other two areas

**Fig. 4:**
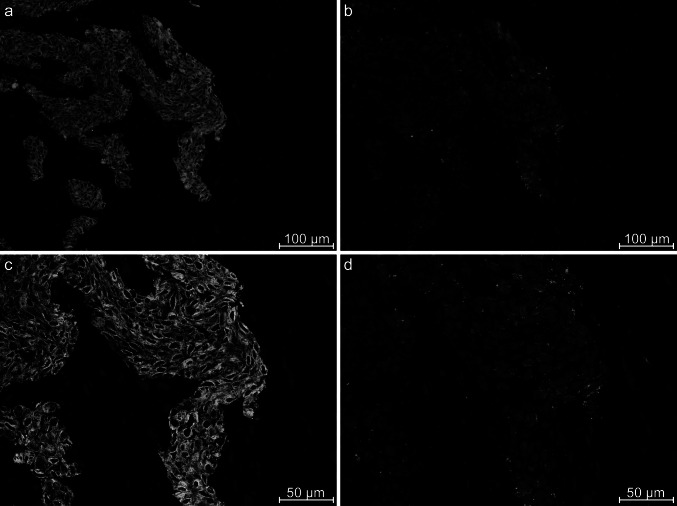
Immunohistochemistry performed on cryostat tissue from a luminal breast cancer of patient 761. All pictures are merged with DAPI staining. The pictures **a** and **c** show staining against CK19. Picture **b** shows staining with LH 7, which only binds a subset of the cancer cells. Picture **d** is the exact same area as in **b**, but in higher magnification. Stainings performed on this biopsy showed a slightly higher frequency of binding to cancer cells, and the staining were generally less grouped compared with staining on the basal-like cancers

IHC was performed on cryopreserved breast tissue from biopsies of four different cancer patients. All tissue sections were co-stained with anti-CK19. This helps to distinguish cancer cells with luminal characteristics from surrounding stroma. The majority of the tested soluble antibody fragments did not show any particular cancer-specific staining, but rather strong staining in the surrounding stroma. However, the antibody fragment LH 7 showed staining toward several minor cell clusters within certain tumor cell nests. This staining pattern could indicate that this particular antibody fragment is associated with certain breast cancer subpopulations. The staining pattern of this antibody fragment does not distinguish between basal-like and luminal cancers. It bound subsets of cancer cells in all of the different cancer biopsies tested. Examples of staining performed on the same basal-like cancer biopsy (P757) as used in the selection are shown in Figs. 2 and 3. Figure 2 shows staining in lower magnification for a more representative view and includes the proliferation control ki67 and a negative control. Figure 3 shows staining in higher magnification on three different areas on the same tissue section (P757). An example of staining performed on a luminal cancer (P761) is shown in Fig. 4. IHC was also performed on breast tissue from three healthy donors, which did not show any binding with LH 7 (Sup. 1).

## Discussion

This paper describes the selection of a domain antibody LH 7, which in repeated immunohistochemistry experiments only recognizes minute cell clusters within certain tumor cell nests. This staining pattern indicates that LH 7 may recognize a breast cancer subpopulation.

The principle of the presented approach relies on the selection of antibody fragments binding to a chosen minute area of breast cancer tissue of interest. This is made possible by the use of phage display and shadow stick. The shadow stick shields the phage particles binding to the target area from UV-C exposure which renders all non-protected phage particles non-replicable in *E*.*Coli*. This approach yields a low output of clones from each selection, while generating a relatively high frequency of phage antibodies binding to unique or upregulated antigens.

Selections were performed on a basal-like breast cancer tissue section with a high-density cluster of CD271^+^ cells within a single cancer nest. These CD271^+^ cells may potentially be classified as breast cancer stem cells [20]. To preserve the structural presentation of antigens, the CD271^+^ target area was identified on a separate slide, and the selections performed on consecutive neighboring slides. As the CD271^+^ cells were targeted within the tumor cell nests on tissue, the screening of recombinant antibodies by phage ELISA was performed on CD271^+^ breast cancer cells, which were single cell cloned from the breast cancer cell line BT474. In the initial phage ELISA screening, 315 phage antibodies from the 13 selections were tested. As CD271^+^ status is common in the myoepithelial cells surrounding tumor cell nests in tissue, these cells were convenient for the purpose of identifying and discarding binders of common epitopes, including binders of the antigen CD271. Thirty-five phage antibodies from the initial screening showed higher absorbance than epsilon and were tested by titration assay on both CD271^+^ cancer cells and CD271^+^ myoepithelial cells. In this assay, it was not the intensity of the absorbance values, which was of interest, but rather the binding difference between the two cell types. These screening and titration assays aided the prioritization of the selected phage antibodies for further evaluation by IHC.

IHC was performed with 11 different soluble antibody fragments on sections from the same biopsy as selected upon to ensure testing on the same tumor microenvironment as they initially bound. Additionally, three other biopsies were used since breast cancer may vary greatly in tumor heterogeneity and phenotype. The IHC experiments were repeated with different batches of purified antibody fragments with similar results. The antibody fragment LH 7 consistently showed binding to subsets of cancer cells within the tumor cell nests in all of the four different cancer biopsies. Hence, this particular antibody fragment recognizes an antigen expressed by a breast cancer subpopulation. The staining performed on the luminal cancer biopsy from patient 761 (Fig. 4) generally showed a higher frequency of staining to the cancer cells, indicating that the subpopulation expressing the cognate antigen of LH 7 may exist in more frequent numbers in this particular biopsy. There were also noticeable differences in the staining pattern in regard to the staining density, both on the same section (Fig. 3) and on another breast cancer subtype (Fig. 4). In some areas, the staining was more clustered in groups of coherent cells (Fig. 3f), and in other areas, it was more scattered around individual cancer cells. Such differences are highly expected due to the heterogeneity of breast cancer.

Immunohistochemistry experiments show that one of the selected antibody fragments, LH 7, is apparently specific toward certain breast cancer subpopulations. Identification and characterization of breast cancer subpopulations may provide new insight and treatment strategies. Further studies identifying and characterizing the subpopulations recognized by LH 7 could be highly valuable for understanding intra-tumor heterogeneity in breast cancer and developing new strategies for diagnosis and treatment. Furthermore, the antibody in itself may possess prospects in the development of reagents for targeted therapy.

## Electronic supplementary material

Supplementary figure 1 Immunohistochemistry on breast tissue sections from three healthy donors for further characterization of LH 7. Picture A, D and G show DAPI staining. B, E and H show staining against CK19. Picture C, F and I show staining with the antibody fragment LH 7. The upper, middle and lower rows represent the healthy donors P671, P820 and P923, respectively. No staining was observed in neither the healthy luminal cells nor stroma (TIFF 5157 kb)
