# Supplementary figures and images for: Selection of a breast cancer subpopulation-specific antibody using phage display on tissue sections
Source: Immunol Res. 2015 May 12;62(3):263–72. doi: 10.1007/s12026-015-8657-x (PMC4469306; doi:10.1007/s12026-015-8657-x)

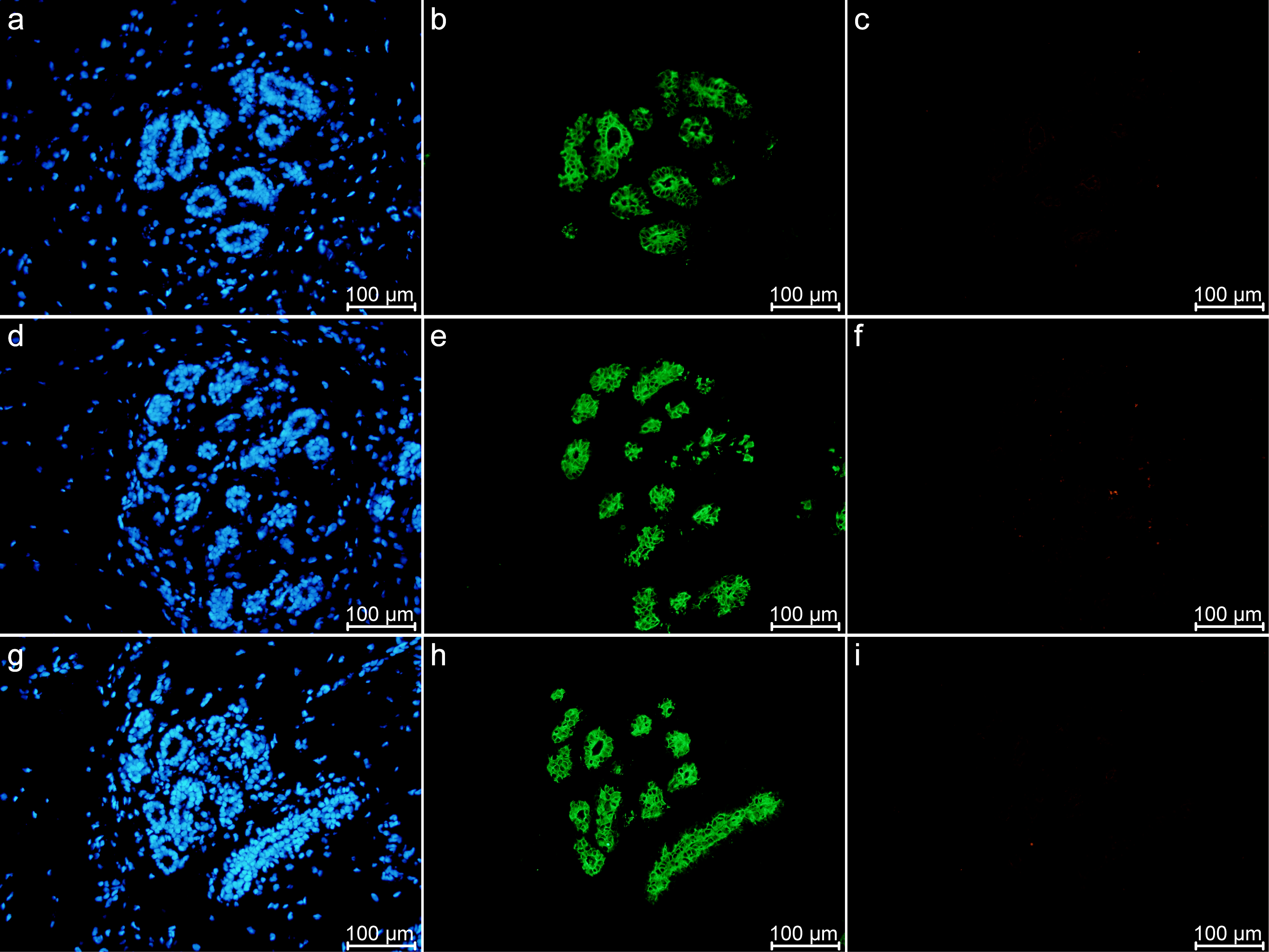

Supplement: Supplementary file 1 — Immunohistochemistry on breast tissue sections from three healthy donors for further characterization of LH 7. Picture A, D and G show DAPI staining. B, E and H show staining against CK19. Picture C, F and I show staining with the antibody fragment LH 7. The upper, middle and lower rows represent the healthy donors P671, P820 and P923, respectively. No staining was observed in neither the healthy luminal cells nor stroma (TIFF 5157 kb) [file 12026_2015_8657_MOESM1_ESM.tif]
